# Stinging Nettle (*Urtica dioica* L.): Nutritional Composition, Bioactive Compounds, and Food Functional Properties

**DOI:** 10.3390/molecules27165219

**Published:** 2022-08-16

**Authors:** Hari Prasad Devkota, Keshav Raj Paudel, Shristi Khanal, Ananda Baral, Nisha Panth, Anjana Adhikari-Devkota, Niraj Kumar Jha, Niranjan Das, Sachin Kumar Singh, Dinesh Kumar Chellappan, Kamal Dua, Philip M. Hansbro

**Affiliations:** 1 Graduate School of Pharmaceutical Sciences, Kumamoto University, 5-1 Oe-honmachi, Chuo-ku, Kumamoto 862-0973, Japan; 2 Headquarters for Admissions and Education, Kumamoto University, Kurokami, 2-39-1, Chuo-ku, Kumamoto 860-8555, Japan; 3 Pharmacy Program, Gandaki University, Pokhara 33700, Nepal; 4 Centre for Inflammation, Centenary Institute and University of Technology Sydney, Faculty of Science, School of Life Sciences, Sydney, NSW 2007, Australia; 5 College of Pharmacy, Yeungnam University, 280 Daehak-ro, Gyeongsan 38541, Korea; 6 Department of Biotechnology, School of Engineering and Technology, Sharda University, Greater Noida 201310, India; 7 Department of Chemistry, Ramthakur College, Badharghat, Agartala 799003, India; 8 School of Pharmaceutical Sciences, Lovely Professional University, Phagwara 144411, India; 9 Department of Life Sciences, School of Pharmacy, International Medical University, Kuala Lumpur 57000, Malaysia; 10 Discipline of Pharmacy, Graduate School of Health, University of Technology Sydney, Ultimo, NSW 2007, Australia; 11 Faculty of Health, Australian Research Centre in Complementary and Integrative Medicine, University of Technology Sydney, Ultimo, NSW 2007, Australia; 12 Uttaranchal Institute of Pharmaceutical Sciences, Uttaranchal University, Dehradun 248007, India

**Keywords:** *Urtica dioica*, nettle, stinging nettle, antioxidant, anti-inflammatory, anti-bacterial, anti-ulcer, nutraceuticals

## Abstract

Stinging nettle (*Urtica dioica* L., Urticaceae) is commonly found in Asia, Africa, and Europe and has a long history of being used as food and traditional medicine. Recently, this plant is gaining attention as a highly nutritious food, where fresh leaves are dried and used as powder or in other forms. Leaves are rich in many bioactive compounds. This review aims to cover the traditional uses in food and medicine, as well as its nutritional composition, including its bioactive chemical constituents and reported food functional activities. Various bioactive chemical constituents have been isolated from stinging nettle to date, such as flavonoids, phenolic acids, amino acid, carotenoids, and fatty acids. Stinging nettle extracts and its compounds, such as rutin, kaempferol, and vitamin A, are also used for their nutritional properties and as anti-inflammatory and antioxidant agents. Future studies should focus on the proper formulation and stability testing of the functional foods containing stinging nettle and their detailed activities in clinical studies.

## 1. Introduction

Medicinal plants have played an important role in human healthcare through their diverse use as food, spices, and traditional medicines, as well as an important source for new drug discovery [1,2]. In recent years, there has been growing interest regarding the plant-based nutraceuticals and functional foods for the prevention of various lifestyle-related diseases and other complications such as inflammation [3,4], hypercholesterolemia [5], atherosclerosis [6], endothelial dysfunction [7], asthma [8] and cancer progression [9,10,11,12,13]. Various plant sources rich in nutritional components such as amino acids, vitamins, and phytochemicals, such as polyphenols, including phenolic acids and flavonoids, are receiving great attention [14,15,16], and many such products are available in different formulations. However, there is a strong need for the identification of active biomolecules (phytochemicals) in these plant products, as well as the detailed study of their pharmacological activities and design of effective formulations and drug delivery systems.

**Figure 1:**
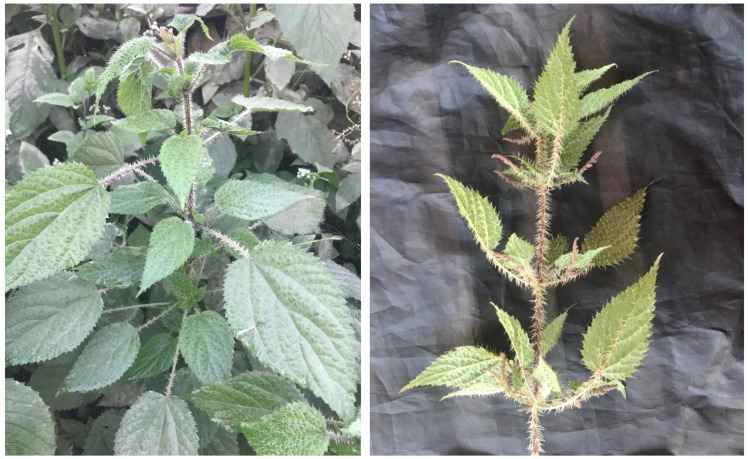
Photographs of *Urtica dioica* (photos by Mr. Prakash Poudel).

*Urtica dioica* L., commonly known as stinging nettle (Figure 1), is a perineal herbaceous plant belonging to the family Urticaceae. It is distributed in temperate region in many parts of the world, including areas in Asia, Europe, North Africa, and North America, up to 1800 m. of altitude [17,18]. In Nepal, it is known as *Sisnu* and is reported to be distributed in moist areas in locations with an altitude of about 500–4500 m [19,20]. The plants are about 2 m in height and covered with stinging hairs with hooked protrusions. The leaves are also covered with stiff hairs on both sides that produce hot sensation when touched [19]. It is well-known for its dermatitis-causing effect when touched, which is mediated by the release of biochemical mediators, such as histamine and acetylcholine from the hairs, which act like needles [18]. It is widely used as a vegetable and after drying as stock food during food shortages [18,19]. One of the well-known uses of *Urtica dioica* is during a process called urtication (external stinging), in which fresh stems and leaves are applied locally to relieve joint pain [21,22]. It has a long history of use as a vegetable and traditional medicine. Various ethnomedicinal studies have reported the use of *Urtica dioica* as a diuretic and for the treatment of cough, cold, cuts, and wounds [19,23,24]. Recently, this plant is gaining attention as a highly nutritious food, where fresh leaves are dried and used as powder or in other forms. The leaves are rich in many bioactive compounds, such as flavonoids, phenolic acids, and amino acids [18]. This review aims to cover the traditional uses as food and medicine, as well as the nutritional composition, including the bioactive chemical constituents, reported pharmacological activities, and recent progress on the formulations of *Urtica dioica*.

## 2. Traditional Uses as Food and Medicine

The plant has been used as nutritious food and traditional medicine for centuries. Leaves and stalks are used for salad and as vegetables, and tea prepared from the leaves, stalks, and roots is also consumed [25]. People of remote hilly regions in Nepal and India collect tender shoots and leaves of this plant, with the help of bamboo or iron pincers, and cook it as a vegetable or soup [19,23,24,26]. It is also boiled with other plants, such as maize, millet, or wheat flour, along with salt and chili to make porridge [19,23]. In recent years, it has been used as a nutritional tonic and for many other preparations, such as dried leaves for herbal tea, or in combination with other herbs, as well as soups, infusions, decoctions, and liquid extracts [27].

In Nepal, the roots are used as a diuretic, astringent, emmenagogue, and anthelmintic agent. Roots are also used for the treatment of cough, cold, jaundice, and asthma [19,20]. Juice of the stems is used for the treatment of fever. Juice of the leaves, obtained by squeezing, is applied to cuts, burns, and wounds. The decoction of the leaves if given to women after childbirth to gain energy, as well as for the treatment of menstrual disorders and jaundice. The paste of leaves is used for the treatment of diarrhea and dysentery. Boiled leaves are used of the treatment of cough and cold [19]. In India, leaf juice is used to treat epilepsy, and it is also used locally to treat boils and blisters [23] It is reported to be used for the treatment of rheumatism and gastrointestinal disorders in Italy [25].

## 3. Nutritional and Chemical Composition

Various species of the genus *Urtica* are reported to be rich sources of nutritional components, such as amino acids, fibers, phenolic compounds, vitamins, and minerals [28]. The leaves of *Urtica dioica* are rich in chlorophylls, carbohydrates, carotenoids, fats, vitamins, and minerals [24,29,30]. Paulauskiene et al. analyzed the influence of harvesting time on the chemical composition of leaves of *Urtica dioica* by collecting the leaves every month from April to September. The results showed a variation in the content of chlorophylls, carotenoids, phenolic compounds, and antioxidant capacity among the samples [29] Adhikari et al. [31] compared the nutritional properties of dried leaf powder of *Urtica dioica* with that of barley and wheat flours. They reported that the leaf powder of *Urtica dioica* had higher level of crude protein (33.8%), crude fiber (9.1%), crude fat (3.6%), and carbohydrates (37.4%), as compared to barley and wheat flours. The energy value was reported to be 307 kcal/100 g. The total phenolic, carotenoid, and tannin contents were 129 mg gallic acid equivalent/g, 3497 μg/g, and 0.93 mg/100 g, respectively.

**Figure 2:**
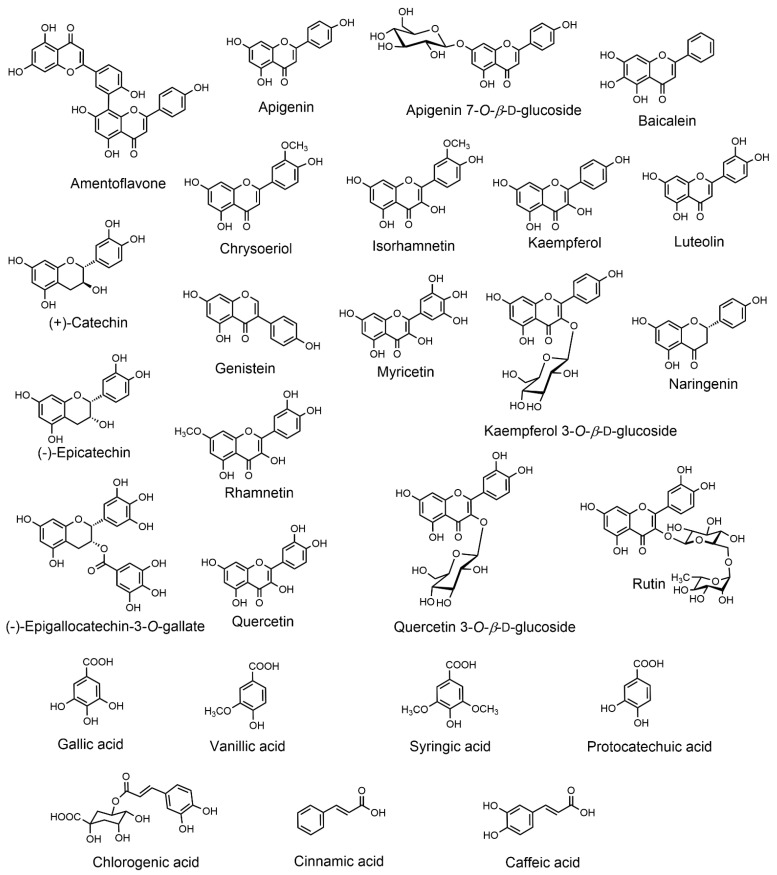
Structures of major flavonoids and phenolic acids reported from the leaves of *Urtica dioica*.

Various phytochemicals, including flavonoids, phenolic acids (hydroxybenzoic acid and cinnamic acid derivatives), amino acids, carotenoids, organic acids, and fatty acids (Table 1) are reported from the different plant parts of *Urtica dioica*, although most of the studies focused on leaves. Structures of some flavonoids and phenolic acids are given in Figure 2. Several compounds of other groups, such as sugars (inositol, glucose, rhamnose, and sucrose) [32], volatile compounds (eg. hexanal, linalool, carvone, cumin aldehyde, carvacrol, and phytol) [33], choline, and indole-3-carboxaldehyde [32], were also reported. Otles and Yalcin reported on the phenolic compounds of the leaves, stalks, and roots of the *Urtica dioica* collected from different parts of Turkey, where they have shown variation in the contents in these compounds based on the plant part and locality from where they were collected [25].

## 4. Food Functional Activities

**Figure 3:**
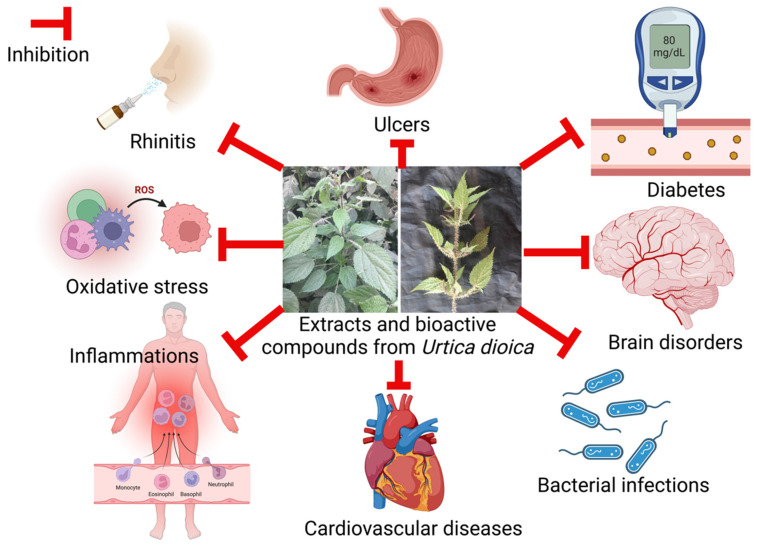
Various food functional activities of extracts and bioactive compounds from *Urtica dioica*. (image created with BioRender.com).

Various food functional activities of extracts and bioactive compounds from *Urtica dioica*, as reported by considerable literature, has been summarized in Figure 3 and Section 4.1, Section 4.2, Section 4.3, Section 4.4, Section 4.5, Section 4.6, Section 4.7 and Section 4.8.

### 4.1. Antioxidant Property

Medicinal plants have been studied widely for their antioxidant properties. An antioxidant shielding system defends cells against the toxic effects of reactive oxygen species (ROS), thereby preventing peroxidation of lipids [37]. The presence of flavonoids and phenolic compounds makes plant become natural antioxidants [38]. For the in-vitro antioxidant test of hydro-alcoholic extract of various aerial parts of *Urtica dioica*, Khare et al. used 2,2-diphenyl-1-picrylhydrazyl (DPPH) free radical scavenging method. For the test, ascorbic acid was used a standard. The result showed that plant extract displayed DPPH activity with IC_50_ value of 88.33 ± 2.88 µg/mL. Ascorbic acid exhibited DPPH activity, with an IC_50_ value of 2.8 ± 0.62 µg/mL. Hence, the result of antioxidant test was positive [39].

Another commonly used method for antioxidant assay is the ferric thiocyanate (FTC) method. Kataki et al. used this method to determine antioxidant property of methanol extract of plant leaves, using α-Tocopherol as a standard. Taking 250 µg/mL concentration, the inhibition of peroxidation of linoleic acid by extract was compared with that of the standard. The result of extract was better than that of the standard, i.e., extract showing 62.34%, while the standard displayed 34.41% [40].

Similarly, Bourgeois et al. used ferric reducing/antioxidant power (FRAP) and the cupric reducing antioxidant capacity (CUPRAC) methods to evaluate the antioxidant activity (relative to antioxidant activity of Trolox at 1 mM) of *Urtica dioica* extract, its active compounds ursolic acid and quercetin. The IC_50_ of CUPRAC was found to be 1.56 ± 0.1, 0.49 ±0.01, and 4.32 ± 0.3, respectively, and the FRAP was found to be 0.47 ± 0.03, 0.14 ±0.03, and 2.51 ± 0.2, respectively [41].

The comparison of antioxidant activity (using FTC methods) of various plants parts of *Urtica dioica* showed that 100 μg/mL hydroalcoholic extracts of the seeds, roots, flower, and leaves showed 81.7%, 79.8%, 78.3%, and 76.4%, respectively. The antioxidant potency (to inhibit lipid peroxidation) was even better than the standard antioxidant BHA, BHT, and *α*-tocopherol, showing 66.2%, 70.6%, and 50.1%, respectively [37].

The antioxidant potential of *Urtica dioica* was also reported by various in vivo studies. Behnam et al. observed that feeding broiler chickens with various dietary levels of *Urtica dioica*, ranging from 0.5–1.5% for up to 6 weeks, results in notable increase in the expression of antioxidant gene catalase (CAT), superoxide dismutase 1 (SOD1) in liver and lung. Moreover, there was significant suppression of lipid peroxidation. The composition of essential oil obtained from Urtica dioica stem and leaves includes various bioactive compounds, such as cadina, copaene, 2-pentyl furan, linalyl acetate, α-terpineol, calamenene, nonanal, β-selinene, cumin aldehyde, eugenol, kessane, limonene, cadinenen, methyl chavicol, pentyl benzene, bisabolene, β-caryophyllene, linalool, furanone, caryophyllene oxide, (E)-geranyl acetone, hexagydrofarnesyl acetone, phytol, anethol, butylidene phthalide, naphthalene, carvone, and carvacol [42]. The expression of antioxidant gene CAT, SOD1, glutathione, and glutathione peroxidase in hippocampal tissue were significantly increased in streptozotocin-induced diabetes Wistar rats fed with daily oral gavage of *Urtica dioica* leaves hydroalcoholic extract (50 mg/kg body weight) for six weeks, compared to diabetes rats without treatment with the extract. Furthermore, the extract also significantly decreased the lipid peroxidation (by measuring the level of malondialdehyde) in hippocampal tissue, compared to diabetes rats [43].

### 4.2. Anti-Inflammatory Property

Herbal medicines exhibit anti-inflammatory activity by targeting various cell signaling pathways or endogenous enzymes, with a key role in inflammation. *Urtica dioica* extracts targets inflammatory cascade-like cyclooxygenase (COX)-1 and COX-2 to inhibit inflammatory prostaglandins and mast cell tryptase to prevent degranulation [44]. The comparison of *Urtica dioica* extract from various plant parts (roots, stems, leaves, and flowers) using various solvents (aqueous, methanol, hexanes, and dichloromethane) for anti-inflammatory activity in the lipopolysaccharide-stimulated murine macrophage cell line (RAW 264.7) showed that activity of dichloromethane extract of roots, stems, and leaves were more potent than other solvent extract in NF-κB luciferase assay. Moreover, the cytotoxicity of dichloromethane extract was minimal in RAW 264.7. This study suggests that lipophilic dichloromethane extract of the roots, stems, and leaves of nettle could be more promising anti-inflammatory than traditional tincture prepared from ethanol, methanol, and aqueous solvent [45]. In an in-vitro study carried by Obertreis et al. the ethanol extract of *Urtica dioica* suppressed TNF-α and IL-1β released in LPS-induced human whole blood. Treatment of a 5 mg/mL concentration of the extract suppressed the level of TNF-α and IL-1β after 24 h of LPS stimulation by 50.8% and 99.7%, respectively [46].

*In-vivo* anti-inflammatory test of *Urtica dioica* was performed by Sabzar et al. by administrating various solvent (hexane, chloroform, ethyl acetate, methanol, and water) extracts of *Urtica dioica* at a dose of 200 mg/kg body weight to Wistar rats. Rat paw edema was induced by administering 1% of carrageenan solution, and indomethacin was used as a standard anti-inflammatory medicine for comparison. Among various solvent extracts, hexane extracts showed significant anti-inflammatory activity by reducing the edema paw volume to 46.51% after 3 h of administration, which was comparable to the activity shown by indomethacin (53.48% reduction in paw edema volume). However, there was no remarkable inhibition of the edema paw volume by methanol, aqueous, and ethyl acetate extract [47]. The protein expression of proinflammatory cytokines TNF-α and IL-1β in hippocampal tissue were significantly decreased in streptozotocin-induced diabetes Wistar rats fed with a daily dose of *Urtica dioica* leaves hydroalcoholic extract (50 mg/kg body weight) for six weeks, compared to diabetic rats without the treatment via extract. This study highlights the potential of *Urtica dioica* extract to attenuate neuroinflammation in the hippocampus [43].

### 4.3. Hypoglycemic Property

Diabetes mellitus arise when blood glucose is not regulated properly in the body, accompanied by increased triglycerides and decreased HDL in blood serum. It is characterized by hyperglycemia (increased in normal blood glucose level) with the symptoms of polyuria, polyphagia, and polydipsia [47]. The administration of hydroalcoholic extract of *Urtica dioica* at doses of 50, 100, and 200 mg/kg/day for 2 weeks to fructose-induced insulin resistance Wistar rats showed a dose-dependent decrease in serum glucose, low density lipoprotein, leptin, and fasting insulin resistance index [48]. Another study of treatment of *Urtica dioica* leaves hydroalcoholic extract in streptozotocin-induced diabetic Wistar rats showed that intraperitoneal injection of extract (100 mg/kg/day) for 5 days significantly reduce the blood glucose concentration (454.7 ± 34.5 in streptozotocin/diabetic rat vs. 303.6 ± 100.6 in extract treatment group) [49]. Bnouham et al. used glucose and alloxan to induce hyperglycemia in rats and evaluated the anti-diabetic activity of aqueous extract of *Urtica dioica*. There were no changes in blood glucose level in alloxan-induced diabetic rats treated with *Urtica dioica* extract; however, in the glucose-induced hyperglycemic rats’ model, 250 mg/kg of aqueous extract administered 30 min before glucose loading showed a remarkable blood glucose lowering effect in rats. It was observed that the extract was able to decrease blood glucose to 33 ± 3.4%, in comparison to the control group after 1 h of glucose administration. These in vivo results provide strong evidence of a remarkable anti-hyperglycemic effect of the *Urtica dioica* extract in rat model [50]. The deficit in insulin sensitivity and signalling factors, such as insulin, the insulin receptor, and the insulin-like growth factor-1 receptor, were improved in streptozotocin-induced diabetic Wistar rats fed with daily dose of *Urtica dioica* leaves hydroalcoholic extract (50 mg/kg body weight) for six weeks, compared to diabetic rats without treatment with the extract [43].

According to an in vitro study by Ranjbari et al., the aqueous extract of *Urtica dioica* leaves at doses of 1.5 and 3 mg/mL markedly induced insulin secretion in pancreatic beta cells (RIN5) cells in a dose-dependent manner. The extract, at the same doses, was also able to stimulate glucose uptake by L6 myotubes, thus showing potent beneficial activity in vitro [51].

### 4.4. Antiulcer Activities

To investigate anti-ulcer activity *Urtica dioica* in ulcers, Gulçin et al. induced ulcer in rats by intraperitoneal administration of ethanol and treated them with 50, 100, and 200 mg/kg of water extract of *Urtica dioica*. Famotidine (20 mg/kg) was taken as a reference drug for the comparison. Measurement of gross lesion in gastric mucosa showed a 34.4% decrease of gastric mucosal injury in famotidine treated groups. *Urtica dioica* water extract showed 67.7%, 61.1%, and 77.8% decrease of mucosal injury at 50, 100, and 200 mg/kg doses, respectively. The results indicated higher antiulcer effect of the extract, compared to that of the reference drug [52]. A comparative study by Burkova et al. investigated the effect of various crushed fragment size (mm to nm) of nettle leaves in prednisolone, acetylsalicylic acid, histamine, and immobilized stress-induced ulcer model. The results showed that the 40–70 nm fragment protects the stomach mucous membrane better than the larger (1 mm) fragment. The antiulcer activity of 40–70 nm fragments was similar to the effect of buckthorn oil. Nettle extracts also inhibit the over secretion of acid and reduced the acidity of stomach juice in an experimental model of peptic ulcer caused by pylorus ligation [53].

### 4.5. Antibacterial Activities

Ghaima et al. studied the antibacterial activity of ethyl acetate extract of *Urtica dioica* against *Salmonella typhi*, *Escherichia coli*, *Staphylococcus aureus*, *Bacillus cereus, and Aeromonas hydrophila*. The well diffusion method was used for the determination of antibacterial activity, using cephalothin as a standard drug. Mueller-Hinton agar was prepared, and bacteria were spread to grow. The introduction of the extract (10 mg/mL), along with the standard (30 µg/mL), was performed to measure the zone of inhibition. The result showed that ethyl acetate extract was effective against all test bacteria. Zone of inhibition of extract against *Aeromonas hydrophila*, *Bacillus cereus*, *Escherichia coli*, *Salmonella typhi*, and *Staphylococcus aureus* were found to be 14, 24, 10, 22, and 20 mm, respectively, whereas the zone of inhibition of cephalothin against those bacteria were observed as 20, 22, 20, 18, and 24 mm, respectively [54].

A similar study was done by Modarresi-Chahardehi et al. against different organisms by treating various *U. dioica* crude drug extracts prepared using hexane, chloroform, ethyl acetate, butanol, diethyl ether, and methanol as a solvent. Extraction was performed using the Soxhlet extractor (Method I) and sequential partitions (Method II). The extracts obtained from ethyl acetate and hexane using method I showed maximum inhibition against pathogenic bacteria, such as *Bacillus cereus*, Methicillin-resistant *Staphylococcus aureus* (MRSA), and *Vibrio parahaemolyticus*. The minimum inhibitory concentration (MIC) values obtained using butanol extract and method II against *Bacillus subtilis* and MRSA were 8.33 and 16.33 mg/mL, respectively; the MIC value for *Vibrio parahaemolyticus* was 0.13 mg/mL using ethyl acetate extract and method II. Moreover, the crude extracts prepared from method I showed better antimicrobial activity against the Gram-positive bacteria than the Gram-negative bacteria [55]. Another study performed by Gulçin et al. showed that aqueous extract of nettle exhibited antimicrobial effects by inhibiting *Escherichia coli, Staphylococcus epidermidis*, *Candida albicans*, and *Proteus mirabilis* with 8 mm zone of inhibition, *Citrobacter koseri, Enterobacter aerogens*, *Streptococcus pneumonia* with 9 mm, and *Micrococcus luteus* with 13 mm zone of inhibition. However, the observed zone of inhibition was less than that of standard amoxicillin-clavulanic acid, micanozole nitrate, ofloxacin, and netilmicin [52]. Zenao et al. studied the antibacterial activity of *U. dioica* extract and its isolated compound in methicillin resistant *Staphylococcu aureus*. Various phenolic acids, flavonoids, flavones, and flavonols were extracted from *U. dioica* leaves. The potential antibacterial activity of *U. dioica* was due to a high content of hydroxycinnamic acids (chlorogenic acid, caffeic acid, and rosmarinic acid) and flavonoid (quercetin) [56].

### 4.6. Cardiovascular-Related Activities

Testai et al. conducted an in-vivo study for hypotensive activity of *Urtica dioica* at various doses in rats. A total of 0.1 mg/kg intravenous administration of extract to anesthetized rats was able to decrease the mean arterial pressure value to 79.5 ± 0.5 mmHg, and 1 mg/kg extract administration decreased it to 65.5 ± 5.5 mmHg. Similarly, 10 mg/kg of extract decreased the mean arterial pressure value to 55.5 ± 9.5 mmHg. The basal value was 96.5 ± 0.5 mmHg. After hypotensive peak, 2–3 min later, the mean arterial pressure became greater and reached initial basal value [57].

Another study conducted by Tahri et al. showed that arterial blood pressure was stable in the control group and decreased progressively. The perfusion of the extract of *Urtica dioica* at 4 mg/kg/h reduced maximally by 15%, which, after the return of the vehicle, increased in recovery period and reached an identical value to the controlled periods. In the second group, during the controlled periods, the arterial blood pressure was stable, but the perfusion of 24 mg/kg/h of extract reduced arterial blood pressure by 38%. Arterial blood pressure increased during recovery period, and the value remained lower, that is, to 30%. In third group, to which the administration of furosemide was performed at a rate of 2 mg/kg/h, arterial blood pressure reduced to 28%. It returned to similar value of controlled period during recovery periods. The acute hypotensive effect of *U. dioica* was due to diuretic and natriuretic effects (excretion of sodium in urine though kidney), suggesting the role of *U. dioica* to modulate renal function [58].

The antihypertensive activity of crude methanolic extract of *Urtica dioica*, as well as its various solvent fraction (ethyl acetate, hexane, chloroform, and aqueous), were explored by comparing the efficacy in normotensive and hypertensive rats. Among various fractions the ethyl acetate fraction showed remarkable antihypertensive activity in hypertensive rats. The ethyl acetate fraction also showed most potent activity of vasorelaxation in rabbit thoracic aortic rings contracted with 80 mM K^+^ and 1 µM phenylephrine. The vasorelaxant activity of ethyl acetate fraction was comparable to reference drug verapamil, suggesting the potential of *U. dioica* in the management of hypertension [59].

### 4.7. Activities Related to Brain Disorders

Neurodegenerative diseases are one of the rising threats to human health and well-being these days. Some of these diseases include Parkinson’s disease, Alzheimer’s disease, Huntington’s disease, etc., as characterized by loss of neuromuscular connection, memory dysfunction, and a generalized systemic neurological defect [60,61,62]. New strategies have been created to combat these diseases.

The role of *U. dioica* in biological field is diverse. Its extract has been studied for its neuroprotective effect in neurodegenerative diseases [63]. A study demonstrated the neuroprotective action of antioxidant rich extract of *Urtica dioica* L. in MPTP model of Parkinson’s disease. The intranigral administration of 1 μM/2μL MPTP on days 1, 7, and 14 caused the depletion of dopamine and its metabolite, as well as an alteration in behavior and reduced dopaminergic cells in the brain. The extract of *Urtica dioica* (UD) was provided by an intragastric route for 14 days at a dosage of 20, 40, and 80 mg/kg by dissolving in sterile water for injection. It improved behavioral performance, motor coordination, and reduced nitrite concentration, when compared to the MPTP group. A total of 80 mg/kg of UD restored the depletion of dopamine and its metabolite and potentiated the neuroprotective effect when administered with minocycline. Furthermore, it also lessened pro-inflammatory cytokines (TNF-α, IL-1β) and restored the glutathione and catalase levels diminished by the administration of MPTP. Collectively, this indicates a significant role of *U. dioica* in the modulation of antioxidant system and ameliorates the damage caused by inflammmatory cytokines [64].

In another study, N-Methyl D-Aspartate (NMDA) lesioned inflammation was induced in Wister rats to mimic neurodegenerative disease, which deteriorated the brain function significantly. Rats subjected to injection of NMDA experienced a reduction in exploring activity and increase in anxiety, whereas exercise, along with a nettle diet, improved the rearing activities significantly (*p* < 0.05, *p* < 0.005). Memory retrieval and learning abilities, estimated by a passive avoidance test, illustrated that NMDA lesion damaged the memory retrieval; on the other hand, nettle supplementation in combination with exercise attenuated lesion induced by NMDA (*p* < 0.05). From the EPR measurement, it was disclosed that supplementation of nettle significantly decreased the accumulation of free radical from cerebellum caused by swimming (*p* < 0.05) [65,66].

The activation of NF-kβ, a transcription factor that leads to oxidative damage via activation of pro-inflammatory cytokines, was found to be substantially inactivated via inhibition of its nuclear translocation by both exercise and nettle supplementation [66].

Ghasemi et al. reported that the hydro alcoholic extract of *Urtica* was neuroprotective in scopolamine-induced memory impairment model. The control group received saline, and 15 mg/kg of scopolamine was injected intraperitoneally in saline in male Wister rats to induce memory impairment. Daily treatment with 20, 50, and 100 mg/kg of *Urtica dioica* was performed for two weeks to assess its protective effect against scopolamine. The Morris water maze (MWM) test results showed that all three doses of *Urtica* were able to reduce the time to reach the platform, compared to scopolamine group (*p* < 0.01–*p* < 0.001). Additionally, in a probe trial test, the scopolamine group lowered the time in target quadrant(Q1), whereas rats administered with scopolamine, along with *Urtica* extract, improved the time spent in target quadrant [67].

### 4.8. Activities Related to Allergic Rhinitis

Cyclooxygenase 1 and cyclooxygenase 2 are enzymes associated with triggering of allergic rhinitis [68]. Similarly, hematopoietic prostaglandin D_2_ synthase (HPDS), which is present in basophils and mast cells and has an important role in inflammatory events in allergic rhinitis [69].

In an in vitro study, the leaf extracts of this plant were shown to inhibit histamine H1 receptor, COX-1, COX-2, HPDS, and tryptase. Authors used GeneBLAzer™ H1-NFAT-*bla* HEK 293T cells and evaluated the effect of *Urtica* extract to H1 receptor. The extract displayed inhibition with IC_50_ value of 251 µg/mL. Tripolidine, a H1 receptor antagonist, used as positive control showed 19 nM IC_50_ value. Likewise, the extract inhibited tryptase with IC_50_ value of 172µg/mL, while tryptase inhibitor protamine achieved IC_50_ value of 103 µM. Cox-1 and Cox-2 inhibitors attained IC_50_ 48 nM and 1.2 µM and the extract achieved IC_50_ of 160 µg/mL and 275 µg/mL [44].

## 5. Clinical Studies on *Urtica dioica*

The root extract of *Urtica dioica* was found to be effective in treating allergic rhinitis. In the study, a double-blind clinical trial was conducted, with 74 patients divided into two groups: placebo and the one treated with 150 mg of *Urtica dioica* for a month. From the sino-nasal outcome test of 40 patients, both groups improved symptoms; moreover, the group receiving the treatment with *Urtica dioica* was able to decrease mean nasal smear eosinophil count significantly, thus implying that *Urtica dioica* could be a supportive therapy for allergic rhinitis [70].

A randomized, double-blind, placebo-controlled trial evaluated the beneficial effects of taking *Urtica dioica* leaf extract at a dose of 500 mg single capsule thrice a day (every 8 h) for 3 months in diabetic patients (*n* = 46), compared with placebo (*n* = 46). As compared to placebo group, the *Urtica dioica* leaf extract was able to significantly reduce the blood levels of fasting glucose, 2 h postprandial glucose, and glycosylated hemoglobin, thus suggesting nettle may improve to control the blood glucose level in type 2 diabetic patients [71].

The mixture of *Urtica dioica* leaves with other herbal formulations has also been investigated for antidiabetic potential. To investigate the antidiabetic activity of mixed herbal formulation of silymarin (obtained from *Silybum marianum* (L) Gaertn (milk thistle) seeds), olibanum (obtained from Boswellia serrata (olibanum gum) resin), and *Urtica dioica* leaves, Khalili et al. conducted a randomized controlled trail with 30 Type I diabetics receiving 1 capsule of herbal formulation (200 mg each of silymarin, olibanum, and nettle leave powder) thrice daily for 90 days (intervention group), as well as 30 Type I diabetic receiving placebo capsule containing 600 mg of toasted powder thrice daily for 90 days. Although there were no significant changes in serum cholesterol or blood pressure among mixed formulation and placebo group, the fasting blood glucose, glycosylated hemoglobin, and triglyceride in the patients receiving mixed herbal drug were notably less than those receiving placebo capsule, suggesting a promising antidiabetic and triglyceride lowering efficacy of the herbal formulation [72].

Another mixture formulation containing *Urtica dioica* leaf extract was proven to be beneficial in patients suffering from gonarthritis (a degenerative disorder of the knee joint). This randomized placebo-controlled trial, compared the efficacy of special formulation called MA212 or Rosaxan, which is mixture of 20 g rosehip (*Rosa canina* L.) fruit puree, 20 g *Rosa canina* fruit juice concentrate, 160 mg *Urtica dioica* leaf extract, and 108 mg devil’s claw (*Harpagophytum procumbens*) root extract in 46 gonarthritis patients *versus* 42 patients receiving placebo [73]. Patients received either 40 mL of MA212 or placebo (40 mL of vegetable juice mixture) once daily after breakfast over a period of 12 weeks. The efficacy of the formulation was calculated based on the Western Ontario and McMaster Universities Arthritis Index (WOMAC), which includes pain, stiffness and function scores, physical and mental quality-of-life scores at 0, 6, and 12 weeks, and analgesic consumption. Although no statistical difference was observed in initial WOMAC scores between formulation vs. placebo groups, during the study, the WOMAC score was improved in formulation group, compared to placebo. The mean pre-post change of the WOMAC pain score was 29.87 in the MA212 group and 10.23 in the placebo group, suggesting a remarkable superiority in favor of MA212.

Similarly, promising efficacy of *Urtica dioica* root extract (UDE) was revealed in another randomized controlled trail investigating the efficacy of UDE on clinical and biochemical parameters in patients with benign prostatic hyperplasia (a urological disorder observed in older male). This comparative study among 30 males in intervention group (450 mg tablet of extract per day for 12 weeks) and 30 males with comparison group (placebo tablet per day for 12 weeks) were evaluated, in terms of international prostate symptoms score (IPSS), serum high-sensitivity C-reactive protein (hs-CRP), malondialdehyde (MDA), and superoxide dismutase (SOD) activity. The results revealed that UDE possesses an intermediate effect on IPSS, while there was a small effect on the serum high-sensitivity C-reactive protein (hs-CRP), intermediate to large effect on malondialdehyde (MDA) levels, and intermediate effect on superoxide dismutase (SOD) activity. In addition, there were no unwanted/side effects observed in any subject after consumption of UDE for 3 months period among BPH patients, which could be beneficial [74].

These considerable clinical studies suggest the potential of *Urtica dioica* alone or in combination with other herbs/nutraceuticals for the management of disease, such as diabetes, arthritic pain, and BPH. More clinical studies are necessary to further validate the clinical efficacy of Urtica dioica in a range of disorders.

## 6. Conclusions and Future Prospects

Young leaves of stinging nettle (*Urtica dioica*) have long been used as nutritional food resources, and various parts are also used as traditional medicines. It is rich in phytonutrients and contains various bioactive phytoconstituents, such as polyphenols. The extracts from *Urtica dioica* have showed potent pharmacological activities such as antioxidative, anti-inflammatory, anti-ulcer, antihyperglycemic, anti-bacterial, and cardiovascular protective activities. However, further research regarding the mechanism of action of bioactive compounds present in *Urtica dioica* is necessary to provide the scientific evidences for its use as traditional medicine and to further develop the extracts and compounds as functional food ingredients. These activities should be supported by detailed clinical studies and proper formulation strategies.

## Figures and Tables

**Table 1:** Bioactive chemical constituents of leaves of *Urtica dioica*.

Chemical Group	Compounds	References
**Flavonoids**	Amentoflavone, apiin, apigenin, apigenin 7-*O*-*β*-d-glucoside, baicalin, baicalein, catechin, epicatechin, epigallocatechin gallate, chrysoeriol, genestein, isorhamnetin, kaempferol, keampferol 3-*O*-*β*-d-glucoside, luteolin, luteolin 7-*O*-*β*-d-glucoside, myrecetin, naringenin, quercetin, quercetin 3-*O*-*β*-d-glucoside, quercetin 3-*O*-*β*-d-galactoside, rutin, vitexin	[25,34,35]
**Phenolic acids**	*Hydroxybenzoic acid derivatives* Gallic acid, vanillic acid, syringic acid, protocatechuic acid, gentisic acid *Cinnamic acid derivatives* Cinnamic acid, caffeic acid, *p*-coumaric acid, ferulic acid, chlorogenic acid, sinapic acid	[25,34,35]
**Amino acids**	Alanine, *γ*-aminobutyric acid (GABA), glutamic acid, isoleucine, leucine, phenylalanine, proline, tyrosine, valine	[32]
**Carotenoids**	*β*-Carotene, lutein isomers, neoxanthin, violaxanthin	[36]
**Organic acids**	Acetic acid, citric acid, formic acid, malic acid, succinic acid	[32]
**Fatty acids**	Arachidic acid, arachidonic acid, behenic acid, dodecendioic acid, euric acid, palmitic acid, palmitolic acid, stearic acid, tricosanoic acid, lauric acid, etc.	[34,36]

## Data Availability

Not applicable.
